# Understanding the effect of temperature and time on protein degree of hydrolysis and lipid oxidation during ensilaging of herring (*Clupea harengus*) filleting co-products

**DOI:** 10.1038/s41598-020-66152-0

**Published:** 2020-06-12

**Authors:** Mursalin Sajib, Eva Albers, Markus Langeland, Ingrid Undeland

**Affiliations:** 1 Food and Nutrition Science, Department of Biology and Biological Engineering, Chalmers University of Technology, SE-41296 Gothenburg, Sweden; 2 Industrial Biotechnology, Department of Biology and Biological Engineering, Chalmers University of Technology, SE-41296 Gothenburg, Sweden; 3 Department of Animal Nutrition and Management, Swedish University of Agricultural Sciences, SE-75007 Uppsala, Sweden

**Keywords:** Sustainability, Biochemistry

## Abstract

The aims of this study were to investigate the effect of temperature, time and stirring on changes in protein degree of hydrolysis (DH), free amino acids (FAA), lipid oxidation and total volatile basic nitrogen (TVB-N) during ensilaging of herring (*Clupea harengus*) filleting co-products. Results showed that temperature and time, and in some cases the interaction effect between these two factors, significantly influenced all the studied responses. Increasing ensilaging temperature and time from 17 to 37 °C and 3 to 7 days, respectively, increased DH, FAA, and TVB-N content from 44.41 to 77.28%, 25.31 to 51.04 mg/g, and 4.73 to 26.25 mg/100 g, respectively. The lipid oxidation marker 2-thiobarbituric acid reactive substances (TBARS) did not increase with time at temperatures above 22 °C, while 2-pentylfuran increased up to 37 °C. Based on the process parameters and responses investigated in this study, and considering energy requirements, it was suggested to perform ensilaging at ambient temperatures (i.e. around 20 °C) with continuous stirring at 10 rpm for 1-3 days; the exact length being determined by the desired DH.

## Introduction

Along with population growth and an increased awareness about the role of protein in a healthy diet, the demand for seafood convenience products is steadily growing^1–6^. As a result, around 70% of all caught/farmed fish is today processed before final sale, in turn generating around 20–80% co-products depending on the type of fish and level of processing^7^. These co-products can be valorized to feed or potentially to food ingredients by ensilaging, which according to the definition of green chemistry proposed by Anastas and Warner^8^, can be described as a “green” process. Ensilaging has several advantages over e.g. fishmeal production such as lower energy and investment requirements, and the flexibility to start the process even in smaller scales at small fish processing sites where fishmeal production is not economically feasible^9–11^. Another important aspect is that an acid preserved ensilaging product (i.e. silage) does not putrefy and is considered as almost sterile^9^. Ensilaging can thus be used to stabilize sensitive fish co-products right after their generation.

The principle of ensilaging is quite simple; minced fish co-products are mixed with organic acids (e.g. formic acid) to lower the pH to a value below 4.0, which preserves co-products against bacterial growth and at the same time induces endogenous protease-mediated autolysis^10^. Several studies have reported positive outcomes in animals using silage in their diet^12,13^, e.g. improved growth performance, immunological status, health and welfare, which is believed to be due to the short-chain peptide contents of silage. Further, the amino nitrogen is more readily absorbed from short-chain peptides (2–6 amino acids) than from the corresponding protein or free amino acid (FAA) mixtures^14–16^; the latter being due to the higher osmotic pressure of FAA than peptides^17,18^. Excessive FAA can also reduce the protein turnover since amino acids are used for energy metabolism instead of protein synthesis^19,20^.

Besides proteins, fish silage also contains polyunsaturated fatty acids (PUFAs) which are highly susceptible to oxidation^21^. An oxidized silage may result in e.g. a lower feed conversion ratio, loss of appetite, and reduced carcass quality as noticed for fish and broilers^9^. Considering upgrading of silage to food, lipid oxidation is indeed also a major factor reducing the sensorial quality. Similarly, high levels of total volatile basic nitrogen (TVB-N), reflecting e.g. formation of volatile amines as trimethylamine nitrogen (TMA-N), can have negative consequences both on the nutritional and sensorial value of silage; the former due to decrease in protein content^22,23^.

Despite these negative consequences of too high levels of FAA, lipid oxidation products and TVB-N^16,24^; very little research has been done to date to optimize the ensilaging process to achieve a desired degree of protein hydrolysis (DH) while keeping the unwanted formation of FAA, lipid oxidation and TVB-N to a minimum. The few studies available on fish silage only comprise analyses of DH and the classic 2-thiobarbituric acid reactive substances (TBARS) to monitor the extent of protein hydrolysis and lipid oxidation, respectively^22,25,26^; however, no studies reported the tuning of the process to minimize negative side reactions. To succeed here, there is a need to deepen current understanding of the ensilaging process and the multiple (bio)chemical reactions it comprises.

One-parameter-at-a-time is the most traditional approach to investigate the effect of process parameters on desired responses. Although this approach is quite simplistic and easy to perform, it is time consuming and lacks the functionality to explore the (potential) interaction effect between parameters. To solve this problem, response surface methodology (RSM) is widely used both in industry and research, which helps to develop a functional relationship between a response and studied parameters, and find the optimum operating conditions^27^. In RSM, Box-Behnken design (BBD) is one of the most commonly used experimental designs which allows to investigate both individual and interaction effect of process parameters in three-level factorial designs, while keeping the number of experimental runs to a minimum^28^. To the best of our knowledge, no previous study has reported on the application of BBD to optimize ensilaging of fish filleting co-products.

The main aims of this study were to: (a) investigate the effect of temperature, time and stirring on DH and lipid oxidation during ensilaging of herring (*Clupea harengus*) co-products, and (b) find an optimum process setting using BBD to achieve a maximum DH while keeping the formation of lipid oxidation, FAA and TVB-N to a minimum. To get detailed information about the protein hydrolysis and lipid oxidation reactions, we applied several different analyses to monitor each of them. The results of this study will guide the producers of fish silage towards a better understanding of the (bio)chemical reactions taking place during the ensilaging process to produce high-quality “silage 2.0” suitable both for feed and for potential food applications.

## Materials and Methods

### Materials

Herring filleting co-products from two different catching seasons were collected from Scandic Pelagic Ellös AB (Ellös, Sweden). The catching seasons were; autumn 2017 (caught in Skagerrak 5807/1012 by the ship Lövön GG778 on 20^th^ August and filleted on 21^st^ August 2017) and spring 2018 (caught in the west part of the Baltic Sea on 25^th^ March by the ships Pollex SM3 and Anna-Lena KÜH24 and filleted on 27^th^ March 2018). The filleting co-products – consisting of around 32% heads, 37% frames, 7% tails, 15% skins, and 9% guts and other intestinal organs – were collected immediately after filleting, transported to the lab in a thermo-box with ice, minced to 2 mm using a meat grinder (la Minerva, Italy), and stored in 500 g aliquots in zip-lock plastic bags at −80 °C until further use. To minimize the influence of batch-to-batch variations on variations caused by our main study parameters, co-products from the autumn 2017 were used for the one-parameter-at-a-time experiments and co-products from spring 2018 in the BBD experiments.

### One-parameter-at-a-time experiments

**Figure 1:**
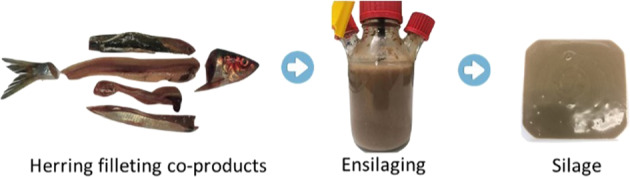
Herring filleting co-products and glass reactors used to produce silage.

One-parameter-at-a-time experiments were first conducted to evaluate the range of parameters for BBD. To investigate the effect of different processing parameters on DH and lipid oxidation, the starting point was a basic ensilaging protocol comprising the addition of formic acid (85% purity) to 500 g minced co-products in a ratio of 2.5% (v/w) in 500-ml glass reactors (Fig. 1). The reactors were continuously stirred at 10 rpm using overhead stirrer and incubated in water baths at 22 °C, unless specified otherwise. Also, effect of endogenous enzymes on DH was studied by inactivating such enzymes at 95 °C for 30 min in a water bath, prior to the incubation at 22 °C. Effect of headspace oxygen removal on DH and lipid oxidation was studied by flushing the headspace of the reactor with N_2_ for 60 secs. Ensilaged samples were collected at different time points and stored in small aliquots at −80 °C until further analysis. A minced herring by-product sample was taken before addition of formic acid (day-0 sample) and was considered as a control. The pH of the day-0 sample was around 6.50, and the pH of ensilaged samples was within the range of 3.42–3.91 during the studied period (Supplementary Information; Fig. 1).

### Multi-response optimization using desirability function

A BBD with six center points was used to investigate the interaction effects of temperature, time and stirring on the responses and to find the optimized process settings to achieve a desired maximum DH while keeping unwanted reactions like lipid oxidation, TVB-N and FAA formation to a minimum. Each process parameter was set on three levels each in the range 17–37 °C, 3–7 days, and 0–20 rpm, respectively. The selected range was based on the observed effect of the parameters on DH and lipid oxidation in the univariate trials, and their relevance for upscale production. Ensilaging was performed as mentioned earlier, and samples were stored in small aliquots at −80 °C until further analysis. Table 1 shows the experimental matrix of the BBD and data for the responses investigated in this study, i.e., DH, FAA, TBARS, malondialdehyde (MDA), 2-pentylfuran, and TVB-N. Experimental design and statistical analysis were performed using primarily Design Expert (version 11, Stat-Ease, Inc., USA), and in some cases MODDE Pro (version 12.1, Sartorius Stedim Data Analytics AB, Sweden) software. Analysis of variance (ANOVA) was used to estimate the statistical parameters, and significant differences were accepted at p < 0.05.

**Table 1:** The experimental design used in the BBD and the responses obtained.

Run	Process parameters			Responses					
	Temp. (°C)	Time (days)	Stirring (rpm)	DH (%)	FAA (mg/g)	TBARS (µmole/Kg)	MDA (µM)	2-pentylfuran (µg/g)	TVB-N (mg/100 g)
1	17	7	10	54.85	39.64	107.20	4.40	23.17	5.46
2	37	3	10	70.04	37.85	90.80	2.75	33.02	9.50
3	27	5	10	66.42	45.93	125.47	4.40	32.15	9.58
4	37	7	10	77.28	51.04	100.73	2.24	52.76	26.25
5	27	5	10	66.11	46.79	105.53	5.48	31.07	9.55
6	17	5	0	44.41	32.70	64.56	2.94	15.35	5.75
7	17	3	10	45.19	25.31	50.49	2.70	14.79	4.73
8	37	5	0	70.67	47.62	54.12	1.50	40.17	20.69
9	27	7	20	76.62	48.96	123.23	4.01	43.68	10.90
10	27	3	0	64.39	35.03	63.24	2.44	27.81	8.06
11	27	7	0	72.81	47.19	59.45	1.76	40.30	13.64
12	27	5	10	66.13	45.81	158.89	3.71	32.33	9.69
13	27	5	10	65.09	46.35	113.45	5.54	32.25	9.58
14	37	5	20	71.96	49.73	94.47	2.45	44.11	11.97
15	17	5	20	49.44	32.44	89.42	3.59	18.85	5.53
16	27	3	20	65.79	33.37	95.27	3.86	31.39	8.07
17	27	5	10	66.48	43.77	169.68	3.60	31.56	9.58
18	27	5	10	65.34	44.81	122.67	4.06	31.60	9.55

Simultaneous optimization of multiple responses was performed using the desirability function^29,30^; individual response was converted into a single desirability function ranging from 0 to 1, where 0 and 1 refer to the least and most desired output, respectively. A high value for DH, and a low value for FAA and the oxidation marker 2-pentylfuran were considered as the most desirable output in this sub-study. The differential weighting function was kept at their default value of 1 for both selected parameters and responses, where a value higher or lower than 1 adds more or less importance, respectively, to that goal during calculation of the multiple desirability function^28^. The settings for the desirability function are given in Table 2.

**Table 2:** Desirability settings for the parameters and the  three selected responses.

Parameters/Responses	Goal	Range	Importance	Weight
Temperature (°C)	Minimize	17–37	+++++ (5)	1
Time (days)	Minimize	1–7	+++++ (5)	1
Stirring (rpm)	Equal to	10	+++ (3)	1
DH (%)	Maximize	30–80	+++++ (5)	1
FAA (mg/g)	Minimize	10–51	++++ (4)	1
2-Pentylfuran (µg/g)	Minimize	0–53	+++++ (5)	1

### Analysis of proximate composition

The moisture content was analyzed by a moisture balance (Precisa HA 300). Total nitrogen content was determined using a nitrogen analyzer (LECO), and then the crude protein content in sample was calculated using a nitrogen to protein conversion factor of 5.58^31^. Crude lipid content was determined gravimetrically using the chloroform phase from a chloroform:methanol (2:1) extraction according to Lee, *et al*.^32^. Ash content was determined by ashing at 575 °C according to NREL protocol (NREL/TP-510-42622)^33^.

### Determination of protein degree of hydrolysis (DH)

DH was determined according to Nielsen, *et al*.^34^ with slight modifications in the sample preparation. Briefly, 0.5 ml silage sample was mixed with 3.75 ml o-phthaldialdehyde (OPA) reagent, vortexed for 5 sec, followed by incubation for 2 min at room temperature. Prior to incubation, silage samples were diluted with pure distilled water to keep the absorbance reading below 1.0, and the absorbance was measured at 340 nm using a spectrophotometer (Cary 60 UV–vis, Agilent technologies, 117 USA).

### Analysis of 2-thiobarbituric acid reactive substances (TBARS)

Silage sample was extracted as mentioned above according to Lee, *et al*.^32^ and the methanol phase was used for TBARS analysis according to Schmedes and Hølmer^35^, with modifications as described by Undeland, *et al*.^36^; the absorbance was measured at 532 nm using a spectrophotometer (Cary 60 UV–vis, Agilent technologies, 117 USA).

### Determination of free amino acids (FAA)

0.9 g silage was weighed in a 1.5-ml Eppendorf tube, centrifuged at 12,000 × g for 10 min (4 °C), then 300 µL supernatant was mixed with an equal volume of TCA solution (7.5% w/v) and kept on ice. The mixture was then vortexed and centrifuged (12,000 × g, 10 min). The supernatant was diluted with 0.2 M acetic acid, and analyzed by LC/APCI-MS according to a method by Özcan and Şenyuva^37^ with slight modifications as described by Harrysson, *et al*.^38^.

### Determination of malondialdehyde (MDA) content

MDA content was determined according to Tullberg, *et al*.^39^, after 2,4-dinitrophenylhydrazine (DNPH) derivatization of samples followed by LC/APCI-MS analysis.

### Determination of 2-pentylfuran content

Volatile 2-pentylfuran was collected using headspace solid-phase microextraction (HS-SPME) technique followed by GC-MS analysis according to a method adopted after Iglesias and Medina^40^ with slight modifications as described by Sajib and Undeland^21^.

### Determination of total volatile basic nitrogen (TVB-N) content

TVB-N was determined using Conway diffusion cells according to Rawdkuen, *et al*.^41^ with slight modifications. Briefly, 2 g silage was mixed with 8 ml of 4% trichloroacetic acid (TCA) solution, vortexed for 2 min, followed by centrifugation at 3,000 × g for 15 min; thereafter, 2 ml supernatant was transferred to the outer ring of the Conway cell. Then, 2 ml of 1% (w/v) boric acid solution containing 0.165% (v/v) methyl red and 0.0825% (v/v) bromocresol green was added to the inner ring of the Conway cell. Subsequently, 2 ml of saturated potassium carbonate solution was added to the outer ring of the Conway cell, and it was covered with the lid, and incubated at 37 °C for 60 min. Upon completion, the inner ring solution was titrated using 0.02 N hydrochloric acid (HCl).

### Analysis of molecular weight distribution of proteins and peptides

Molecular weight distribution of samples was analyzed using HP-SEC according to Abdollahi, *et al*.^42^ with the modification that 5 g of silage was diluted with 5 ml of mobile phase prior to centrifugation.

### Statistical and multivariate analysis

The results were expressed as mean (n = 3) ± standard error of the mean (SEM). Statistical analysis was performed on R (https://www.r-project.org/). The obtained results were subjected to either one-way or two-way ANOVA, and comparison among samples was performed with Tukey Honest Significant Differences (HSD) test with p < 0.05 representing a significant difference. Multivariate analysis was performed using SIMCA software (version 15, Umetrics, Sweden).

## Results and Discussion

### Proximate composition of herring filleting co-products

The composition of herring filleting co-products from autumn and spring is shown in Table 3. The main difference was in the lipid and water contents, which are normally inversely related to each other. The lower lipid content in spring than autumn is in line with earlier reports on low lipid content prior to the spawning season^43^. The protein and ash content were however more or less the same irrespective of the catching season.

**Table 3:** Proximate composition of herring filleting co-products.

Sampling time	Moisture (%)	Crude protein (%)	Crude lipid (%)	Ash (%)	Others (%)
Autumn 2017	66.70 ± 0.14^*^	12.29 ± 0.15^*^	17.85 ± 0.05^*^	2.81 ± 0.10	0.35
Spring 2018	77.03 ± 0.19^*^	14.95 ± 0.08^*^	4.55 ± 0.21^*^	2.69 ± 0.05	0.78

Star sign represents significant (p < 0.05) difference between the two seasons. Results are expressed as mean ± SEM (n = 3).

### Effect of processing parameters on DH and lipid oxidation

**Figure 2:**
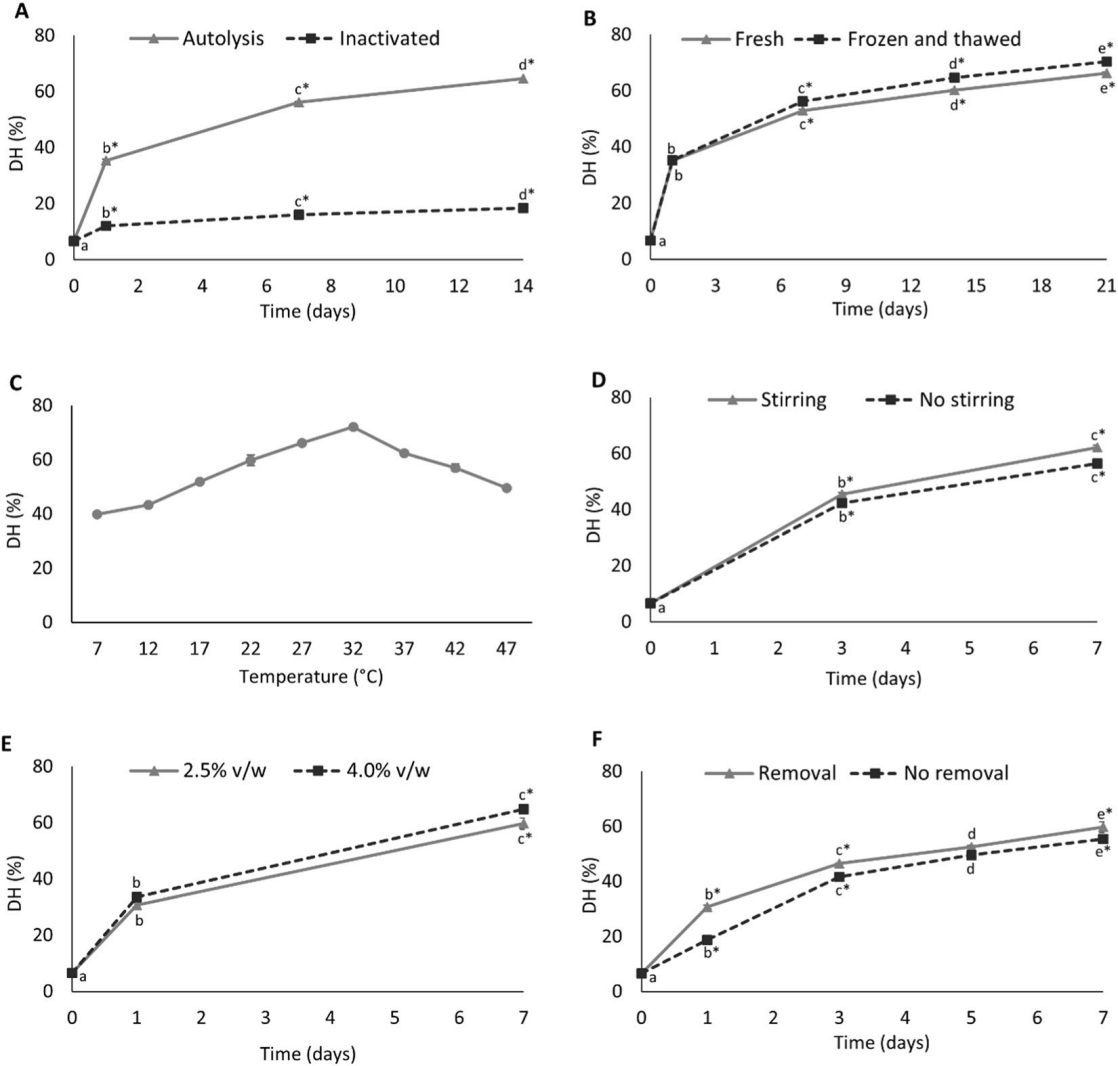
Effect of different parameters on protein degree of hydrolysis (DH). The basic protocol used, if not varied as stated below, was addition of 85% formic acid at a 2.5% (v/w) level, ensilaging at 22 °C with stirring at 10 rpm but without oxygen removal. The different panels show the effect of e.g. (**A**) inactivating enzymes at 95 °C for 30 min vs autolysis, (**B**) time, (**C**) varying temperature, (**D**) stirring, (**E**) acid to fish co-product ratio, (**F**) headspace oxygen removal with N_2_. Data points along the same line with different lower-case letter are significantly (p < 0.05) different from each other; star (*) sign represents significance (p < 0.05) difference between treatments at different time points. Results are expressed as mean ± SEM (n = 3).

The increase in DH over time was mainly due to autolysis since only a very small, but significant (p < 0.05), increase in DH was noticed after inactivation of endogenous enzymes at 95 °C for 30 min (Fig. 2A). In addition, DH was clearly time dependent and increased up to around 7 days where after the rate levelled off (Fig. 2B); similar observation was also reported by van’t Land, *et al*.^22^. Further, a slightly but significantly (p < 0.05) higher DH was noticed when frozen and thawed co-products were used as opposed to fresh ones, which could be due to the fact that cell membranes were disrupted due to ice crystal formation facilitating a better contact between endogenous enzymes and the fish proteins. In Fig. 2C, DH after 7 days of ensilaging is shown as a function of temperatures beyond that of our basic protocol (22 °C); the maximum DH was found at 32 °C, which means that the endogenous proteolytic enzymes of herring co-products were most active at around this temperature. A slight but significant (p < 0.05) increase in DH was noticed both after 3 and 7 days when a continuous stirring was used, compared to when stirring only was done during the 30 first minutes and prior to sampling to ensure homogeneity (Fig. 2D). The difference was surprisingly small, indicating an efficient diffusion of enzymes/substrates even without a continuous stirring. A slight but significant (p < 0.05) increase in DH was noticed after 7 days of ensilaging when 4% (v/w) formic acid of 85% purity was used (Fig. 2E), compared to a 2.5% (v/w) addition, which could be due to an increased contact between enzymes and proteins due to lower viscosity. However, addition of a higher level of acid will increase the moisture content of the final silage; hence, will increase drying cost if aiming at a dried silage product. Further, a 2.5% (v/w) addition of acid was sufficient to bring the initial ensilaging pH to around 3.50, the reported optimum pH for digestive proteases from herring^9,44^, and this level also allows direct addition into a feed product without neutralization of the silage^45^. In all subsequent ensilaging experiments, 2.5% (v/w) acid was therefore added. Figure 2F shows the effect of removing oxygen from the ensilaging reactor’s headspace on DH. An incentive for doing so could be to suppress lipid oxidation. The observed slightly lower DH progression without headspace oxygen removal was somehow different than earlier findings^46^, where Dosoretz, *et al*.^46^ reported increased protease activity with increased oxygenation.

**Figure 3:**
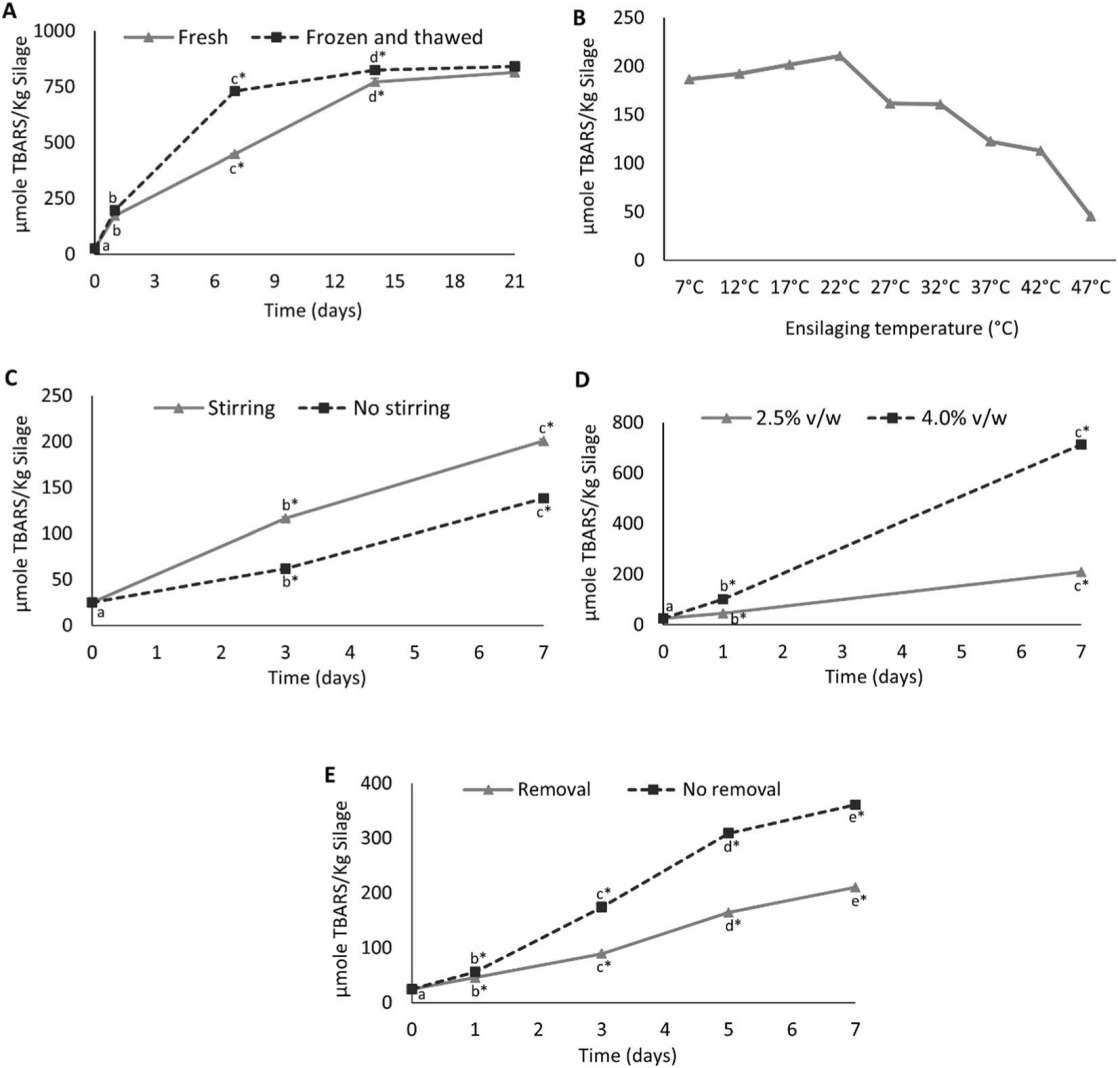
Effect of different parameters on lipid oxidation (TBARS). The basic protocol used, if not varied as stated below, was addition of 85% formic acid at a 2.5% (v/w) level, ensilaging at 22 °C with stirring at 10 rpm but without oxygen removal. The different panels show the effect of (**A**) time, (**B**) temperature, (**C**) stirring, (**D**) acid to fish co-product ratio, and (**E**) effect of headspace oxygen removal with N_2_. Data points along the same line with different lower-case letter are significantly (p < 0.05) different from each other; star (*) sign represents significance (p < 0.05) difference between treatments at different time points. Results are expressed as mean ± SEM (n = 3).

Figure. 3 shows how the variation in process parameters affected TBARS as a marker of lipid oxidation. TBARS increased steadily over time when ensilaging was performed at 22 °C (Fig. 3A). However, the rate levelled off after 6 and 14 days, which could be explained by the fact that Hb/myoglobin (Mb) becomes limiting for the peroxide breakdown^47^ and/or that carbonyls reacted further with e.g. proteins, peptides and amino acids, resulting in tertiary lipid oxidation products^21,48^. Similar to our findings, van’t Land, *et al*.^22^ also reported a decreasing trend of TBARS over time. The use of frozen and thawed co-products resulted in relatively higher level of TBARS, compared to fresh co-products, which could possibly be due to disruption of cell wall structures enabling the PUFAs more accessible to attack by pro-oxidants and free radicals. Further, TBARS decreased with increasing temperature (Fig. 3B), possibly because the main carbonyl compound responding in TBARS test i.e. MDA did not accumulate but rather reacted further when ensilaging was performed above ambient temperatures (i.e. >22 °C)^21,24^. Stirring resulted in a significantly (p < 0.05) higher TBARS values, compared to stirring only at start and prior to sampling (Fig. 3C); probably due to increased oxygen level in the system. A high acid to co-product ratio resulted in a significant (p < 0.05) increase in TBARS after 7 days of ensilaging (Fig. 3D), which is most probably due to enhanced mobility of reactants in the system due to the decreased viscosity and increased water activity^49^, as well as to the fact that a lower pH further activated heme-proteins as pro-oxidants^50^. Figure 3E shows that the removal of headspace oxygen resulted in significantly (p < 0.05) lower levels of TBARS, which was an expected finding. Thus, limiting the oxygen supply to the system could be implemented if targeting a high-quality silage. This procedure would however have to be weighed carefully against increased production costs and was not implemented as a standard procedure in the current study.

**Figure 4:**
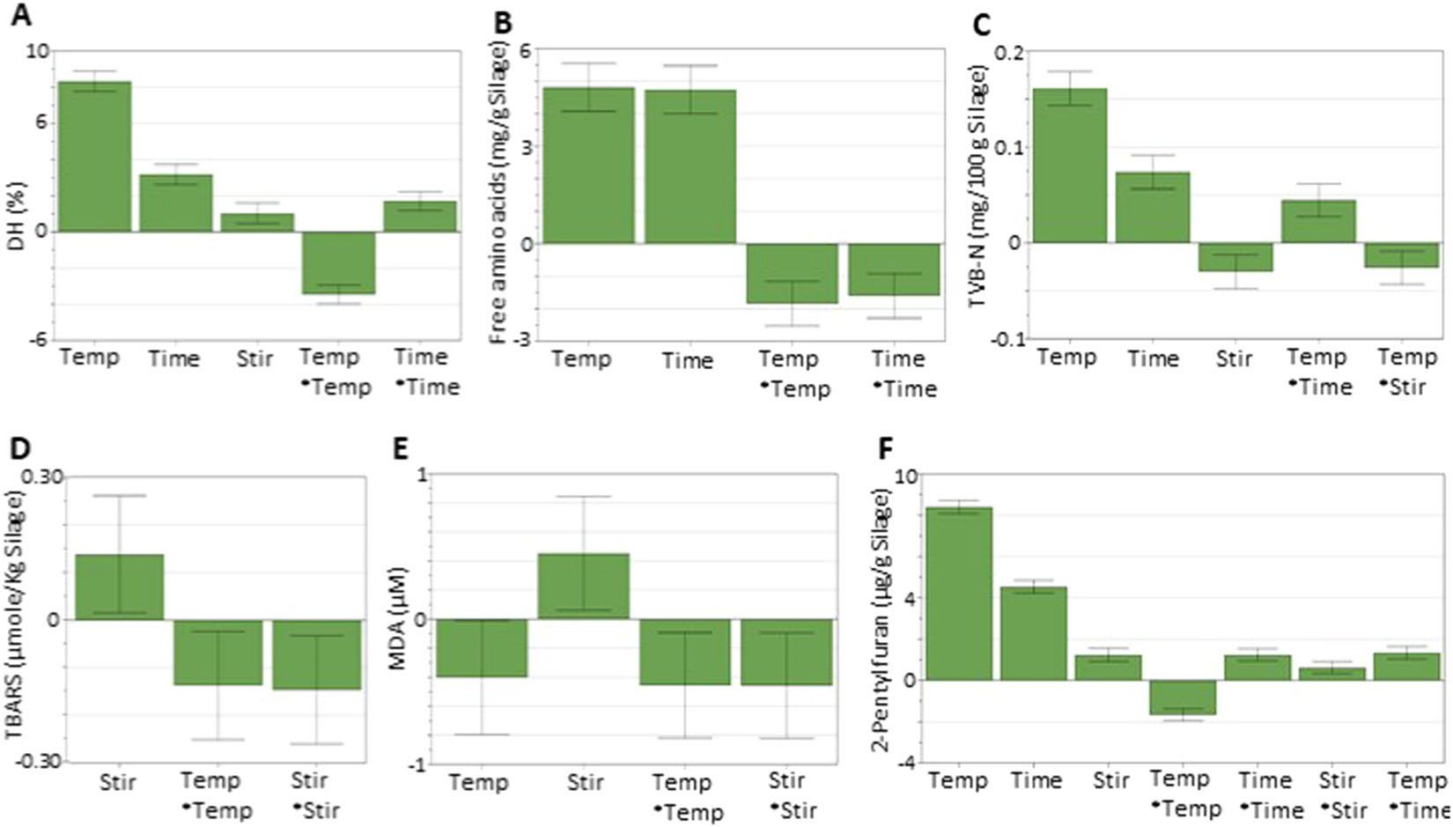
Coefficient plots showing the significant (p < 0.05) model terms (i.e. regression coefficients) from evaluation of the BBD with error bars representing confidence intervals; the different panels show the model terms for (**A**) DH, (B) FAA, (**C**) TVB-N, (**D**) TBARS, (**E**) MDA, and (**F**) 2-pentylfuran. Responses were scaled and centered, and the size of coefficients represents the change in their respective responses when a factor varies from medium to high level (see Table 1), while keeping other factors at their average values.

### Optimization of the ensilaging process

Considering the documented influence of process parameters on both DH and lipid oxidation in the univariate trials, it was decided to perform an experimental design (i.e. a BBD, see Table 1) in which temperature (17–37 °C), time (3–5 days), and stirring (0–20 rpm) were varied to find out an optimum operational setting with respect to high DH but minimal lipid oxidation and FAA; also keeping process scalability in mind. Based on the results of Fig. 3B, which is along with results of our recent study^21^, lipid oxidation was here monitored by specific analyses of MDA and 2-pentylfuran, in addition to the TBARS test.

**Figure 5:**
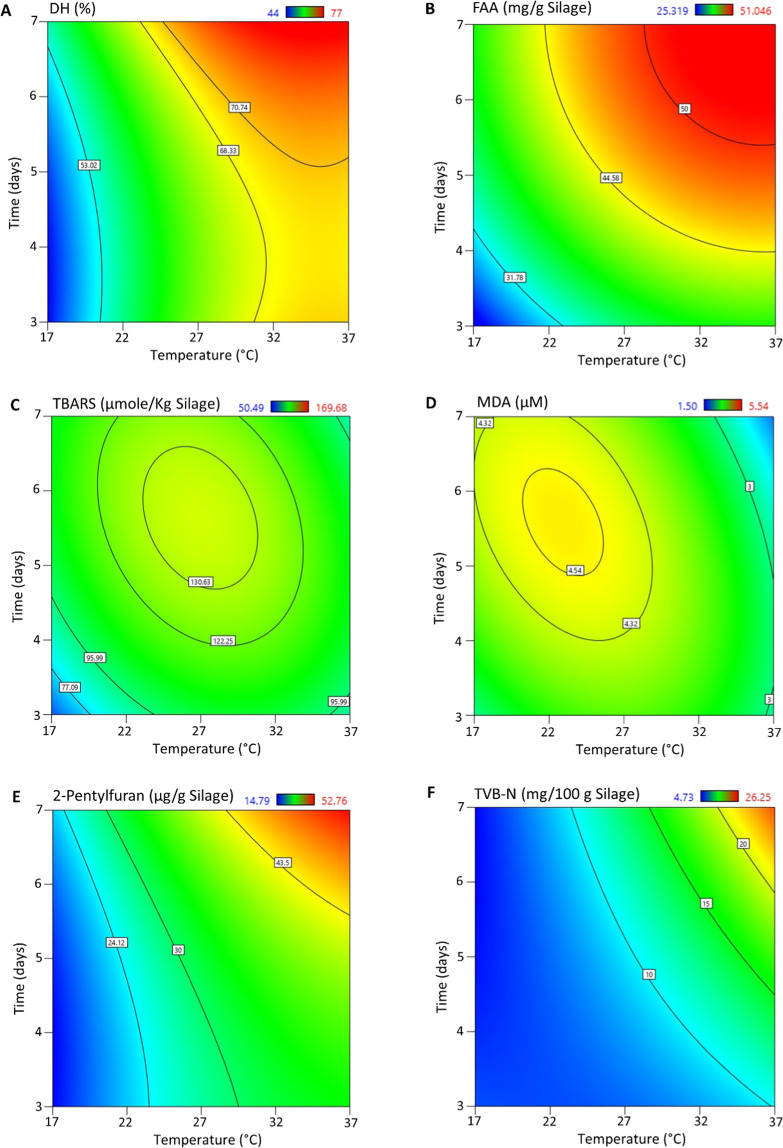
Contour plots showing the effect of temperature and time on (**A**) DH, (**B**) FAA, (**C**) TBARS, (**D**) MDA, (**E**) 2-pentylfuran and (**F**) TVB-N when evaluating the BBD. Stirring was set at 10 rpm for all responses.

**Figure 6:**
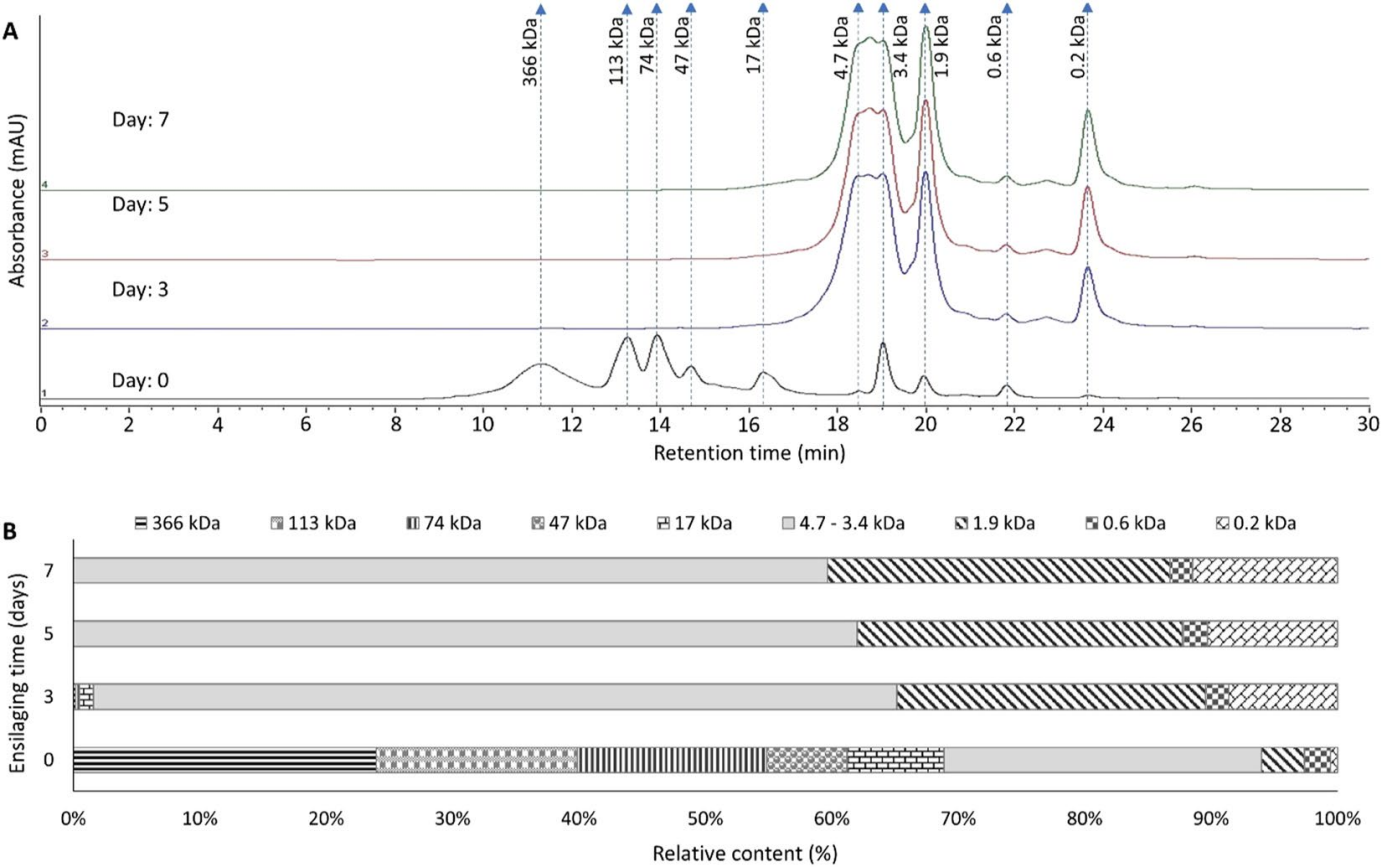
Changes in molecular weight distribution of proteins and peptides over time (0–7 days) at 27 °C; (**A**) SEC chromatogram, (**B**) relative content of differently sized proteins and peptides.

Based on the ANOVA analysis (see Supplementary Information; Tables 7–12), the regression models for all six studied responses were significant (p < 0.05). Further, the lack-of-fit values were not significant (p > 0.05) for studied responses, except for TVB-N, suggesting that the model fitted the data very well^51^. The fit of the models was also evaluated by adjusted R^2^ values, which minimizes the possibilities of overfitting the models, where a value close to 1.0 represents a good fit of the model^52,53^. The adjusted R^2^ values for the quadratic models of DH, FAA, and 2-pentylfuran were 0.99, 0.96, and 0.99, respectively, suggesting a very good fit of the models. This also resulted in a very high predicted R^2^ values, a measurement of the predictive quality of the model, of DH, FAA, and 2-pentylfuran (see Supplementary Information; Tables 1–6, and Fig. 5). The adjusted R^2^ values for the quadratic models of TBARS, MDA, and TVB-N were in the range of 0.56–0.92, however, the predicted R^2^ values were quite low (in the range of 0.10–0.43); therefore, only the responses DH, FAA, and 2-pentylfuran were considered for multi-response optimization of the ensilaging process using the desirability function. Further, the normal plot of residuals for all six studied responses was linear (see Supplementary Information; Fig. 6), suggesting that none of the responses deviated from normality^54,55^.

The effects of temperature, time, and stirring on studied responses were evaluated through regression analysis, and the resulting quadratic equations for responses are given in Eqs. 1–6, where A = temperature, B = time, and C = stirring. A positive coefficient value indicates that an increase in that process parameter will result in an increase in that response, and a negative coefficient will influence the result negatively. Figure 4 shows the model terms which had significant (p < 0.05) effects on the responses and reveals that the most important model terms in most cases, were temperature and time, and in some cases stirring. Also, interaction effects between two of the three terms temperature, time, and stirring had significant (p < 0.05) effects on some of the responses e.g. TVB-N, 2-pentylfuran; however, the main effects were always higher than the interaction effects (see Supplementary Information; Table 7–12).

1 $${\rm{DH}}=65.67+12.12{\rm{A}}+4.62{\rm{B}}+1.50{\rm{C}}\,-\,0.75{\rm{AB}}\,-\,1.00{\rm{AC}}+0.50{\rm{BC}}\,-\,7.46{{\rm{A}}}^{2}+3.54{{\rm{B}}}^{2}+0.79{{\rm{C}}}^{2}$$

2 $${\rm{FAA}}=45.58+7.02{\rm{A}}+6.91\,{\rm{B}}+0.24\,{\rm{C}}\,-\,0.28\,{\rm{AB}}+0.59\,{\rm{AC}}+0.85\,{\rm{BC}}\,-\,3.82\,{{\rm{A}}}^{2}\,-\,3.30\,{{\rm{B}}}^{2}\,-\,1.14\,{{\rm{C}}}^{2}$$

3 $${\rm{TBARS}}=132.62+3.56{\rm{A}}+11.35\,{\rm{B}}+20.13\,{\rm{C}}\,-\,11.69\,{\rm{AB}}+3.87\,{\rm{AC}}+7.94\,{\rm{BC}}\,-\,27.48\,{{\rm{A}}}^{2}\,-17.83\,{{\rm{B}}}^{2}\,-\,29.49\,{{\rm{C}}}^{2}$$

4 $${\rm{MDA}}=4.47\,-\,0.58A+0.08\,{\rm{B}}+0.65\,{\rm{C}}\,-\,0.55\,{\rm{AB}}+0.07\,{\rm{AC}}+0.20\,{\rm{BC}}\,-\,0.92\,{{\rm{A}}}^{2}\,-\,0.52\,{{\rm{B}}}^{2}\,-\,0.92\,{{\rm{C}}}^{2}$$

5 $$2 \mbox{-} {\rm{pentylfuran}}=31.83+12.24{\rm{A}}+6.61\,{\rm{B}}+1.80\,{\rm{C}}+2.84\,{\rm{AB}}+0.11\,{\rm{AC}}\,-\,0.05\,{\rm{BC}}-3.53\,{{\rm{A}}}^{2}+2.64\,{{\rm{B}}}^{2}+1.33\,{{\rm{C}}}^{2}$$

6 $${\rm{TVB}} \mbox{-} {\rm{N}}=9.59+5.87{\rm{A}}+3.24\,{\rm{B}}\,-\,1.46\,{\rm{C}}+4.01\,{\rm{AB}}\,-\,2.13\,{\rm{AC}}\,-\,0.68\,{\rm{BC}}+1.36\,{{\rm{A}}}^{2}+0.53\,{{\rm{B}}}^{2}+0.03\,{{\rm{C}}}^{2}$$

**Figure 7:**
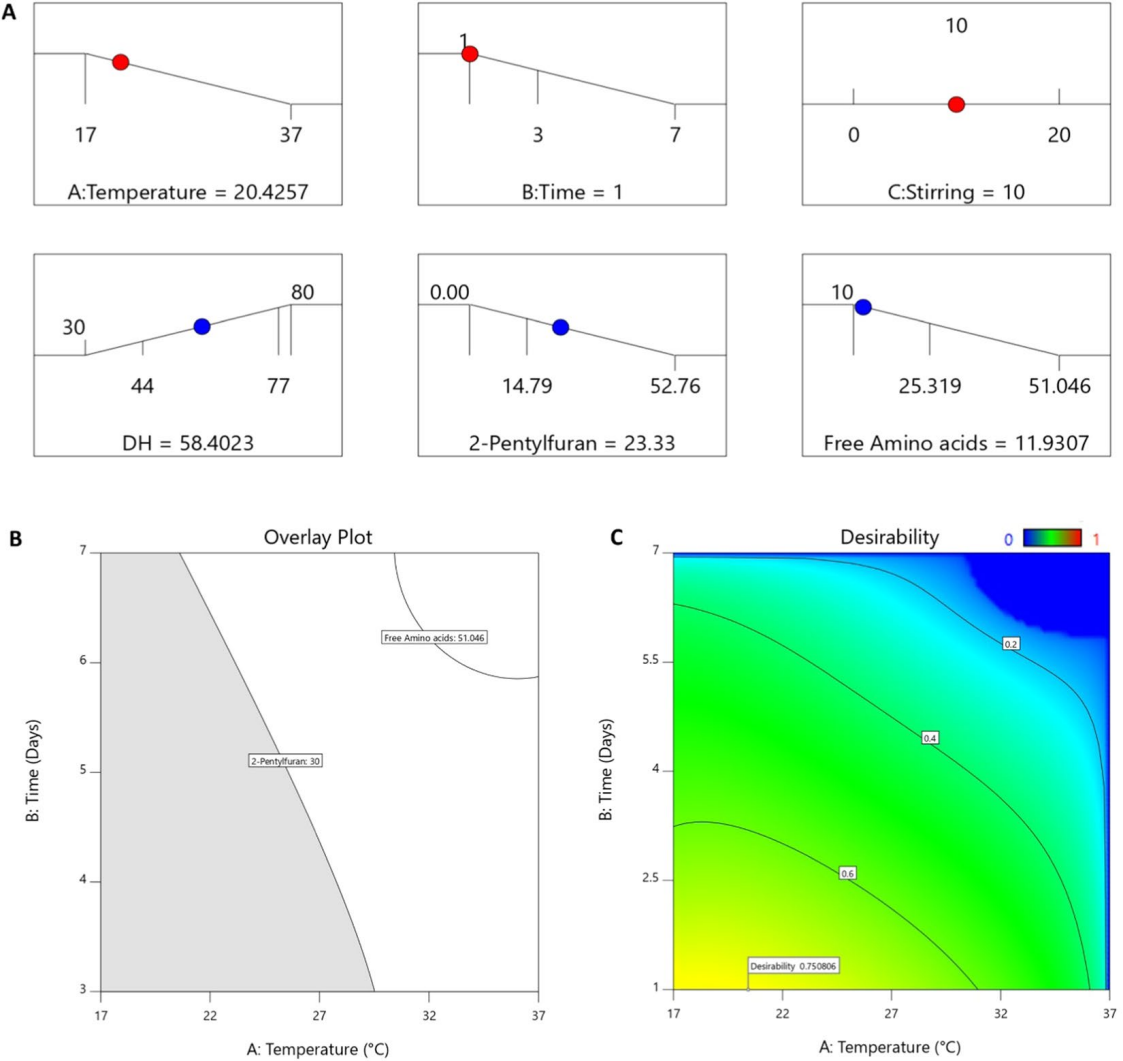
Graphical representation of (**A**) desirability ramp: the optimum value of temperature, time, and stirring to achieve possible maximum DH of 58% while keeping the 2-pentylfuran and FAA content to a minimum with an overall desirability score of 0.75 out of 1.0, (**B**) overlay plot showing suggested ensilaging settings (grey region) within investigated parameter settings in this study at 10 rpm stirring, and (**C**) contour plot of desirability function for optimum ensilaging settings mentioned in the desirability ramp: effect of temperature and time at 10 rpm stirring.

It is evident from Fig. 5A that DH increased with temperature and time up to 37 °C and 7 days, respectively. The observed increase in DH even after 32 °C was somewhat different from the results of Fig. 2C, in which herring co-products from the autumn season were used, indicating that the endogenous proteolytic enzymes present in herring co-products collected in the spring season were most active above 32 °C. The optimum activity temperature of herring muscle protease has been reported to be around 50 °C at pH 3.80^56^; supporting that increased DH at temperatures above 32 °C is still within a natural range for herring proteases. The FAA content also increased with both temperature and time (Fig. 5B), and correlated strongly with DH (see Supplementary Information Figs. 3–4), which has also been reported by other authors^57^. However, besides FAA, peptides were also produced over time during the ensilaging process, and, the relative content of peptides was around 90% (Fig. 6). Most of the proteins with sizes between 17–366 kDa were hydrolyzed into peptides mainly within the size of 1.9–4.7 kDa within 3 days of ensilaging. The observed peak around 0.2 kDa could possibly be due to FAA and/or small di-peptides^58^, which increased over time. Therefore, to produce a high-quality silage, both DH and FAA should be considered with the aim to increase the DH while keeping the FAA content to a minimum. Looking into the specific FAA released, no specific pattern was noticed in this BBD study (see Supplementary Information; Figs 7–8).

TBARS and MDA (Fig. 4D,E, respectively) responded most different to the model terms in that temperature and time had very little influence on their formation. This is also evident when looking at their respective contour plots shown in Fig. 5C,D. As can be seen here, both TBARS and MDA accumulated around 22–27 °C (within 5–6 days), then degraded at elevated temperatures. MDA, being the main carbonyl responding in the TBARS test, can be subjected to hydrolytic cleavage at elevated temperature to form acetaldehyde and formic acid^21,24^. It can also react further with e.g. proteins/peptides/amino acids to give rise to the formation of non-enzymatic browning reaction products^21,48^. Hence, lower TBARS and MDA values at elevated temperature does not necessarily imply less oxidation, but just a different profile of oxidation-derived products^21^. This theory was confirmed by the fact that 2-pentylfuran – which can be formed both from the oxidation of n-3 LC PUFAs and from non-enzymatic browning reactions – followed an increasing trend with both temperature and time alone, as well as with their interaction effect (Figs. 4F and 5E). Thus, if ensilaging is done >22 °C, it is important that the correct oxidation markers are used if aiming at a high quality fish silage and/or at extracting high-quality oils for feed or human consumption^26^. For the latter, lipid oxidation should be kept within the acceptable limits for edible oils set by the GOED Voluntary Monograph^59^.

Similar to 2-pentylfuran, TVB-N followed an increasing trend with both temperature and time (Fig. 5F) which was in agreement with an earlier study on fish silage^22^ and may result from the extended hydrolysis (Fig. 5A) with subsequent deamination of amino acids containing amide-N groups, e.g. glutamine and asparagine, into NH_3_^22^. TVB-N is a freshness indicator often applied at different steps during fishmeal and fish silage production. A value below 50 mg/100 g refers to a good-quality fishmeal^22^; while no such limits are yet described for fish silage.

### Multi-response optimization using the desirability function

Among the six responses studied in the BBD, DH, FAA, and 2-pentylfuran were selected to find the optimum process settings with the aim to maximize the DH while keeping the unwanted formation of FAA and 2-pentylfuran to a minimum. TVB-N can also be used as a freshness indicator of fish silage; however, it was not considered in this study due to significant (p < 0.05) lack-of-fit and low predicted R^2^ values of the model (see Supplementary Information; Table 6 and 12). It was further decided to minimize the ensilaging temperature and time, to perform the ensilaging in an energy-smart manner, while at the same time produce a high-quality silage. The desirability settings of multi-response optimization are given in Table 2, and the outcomes of this desirability settings are shown in Fig. 7. Temperature and time were found to influence the ensilaging process more than stirring; thus the importance score of 5 was given to both temperature and time while the stirring was kept constant at 10 rpm to secure proper mixing of acid with minced co-products to avoid microbial spoilage (Table 2). Based on the inputs given on the desirability function (see Table 2), ensilaging should be performed within the temperature range of 17–30 °C for 3–7 days as shown in the overlay plot in Fig. 7B. However, based on the model prediction, it was further recommended to perform ensilaging at ambient temperature (i.e. ∼20 °C) for 1–3 days (Fig. 7C); the exact duration of the ensilaging being depending on the desired DH. The role of the latter for the value of silage as an aquafeed ingredient needs further evaluation. It is generally accepted that smaller peptides are more bioavailable to fish than larger ones^60–62^, but the difficulty in controlling the enzymatic activity during autolysis makes it difficult to maximize the formation of small peptides yet avoiding FAA formation. The latter has, as earlier stated, been shown to lower the nutritional and biological value, and increase fluctuations in plasma amino acid levels^14,16,63^.

## Conclusions

The effect of temperature and time on (bio)chemical reactions taking place during ensilaging of herring co-products has here been systematically investigated and reported for the first time. Temperature and time were the most significant parameters influencing the studied responses (i.e. DH, FAA, TBARS, MDA, 2-pentylfuran, and TVB-N); however, in some cases the interaction effects of these two parameters also influenced the responses significantly. At temperature >22 °C, 2-pentylfuran was a better lipid oxidation marker for silage than TBARS/MDA. It was generally found that the formation of FAA, lipid oxidation products and TVB-N increased with time, which is expected to lower the value of the silage as a feed/food ingredient. Using a multivariate approach comprising a BBD it was seen that performing ensilaging at ambient temperatures (i.e. ∼20 °C), with continuous stirring at 10 rpm for 1–3 days produces the highest quality silage and keeps energy input levels at a minimum. The exact ensilage time will depend on the desired DH; the latter being an area that needs further research.

## Supplementary information

Supporting information.
