# Supplementary material for: Understanding the effect of temperature and time on protein degree of hydrolysis and lipid oxidation during ensilaging of herring (Clupea harengus) filleting co-products
Source: Sci Rep. 2020 Jun 12;10:9590. doi: 10.1038/s41598-020-66152-0 (PMC7293326; doi:10.1038/s41598-020-66152-0)
Supplement: Supplementary file 1 — Supporting information. [file 41598_2020_66152_MOESM1_ESM.docx]

**Supplementary Information**

**Understanding the effect of temperature and time on protein degree of hydrolysis and lipid oxidation during ensilaging of herring (*Clupea harengus*) filleting co-products**

Mursalin Sajib^a^*, Eva Albers^b^, Markus Langeland^c^ and Ingrid Undeland^a^

^a^Food and Nutrition Science, Department of Biology and Biological Engineering, Chalmers University of Technology, SE-41296 Gothenburg, Sweden

^b^Industrial Biotechnology, Department of Biology and Biological Engineering, Chalmers University of Technology, SE-41296 Gothenburg, Sweden

^c^Department of Animal Nutrition and Management, Swedish University of Agricultural Sciences, SE-75007 Uppsala, Sweden

*Corresponding author

Tel: +4631 772 68 63

E-mail: [mursalin@chalmers.se](mailto:mursalin@chalmers.se)

Table 1. DH – model fit summary

| **Source** | **Sequential p-value** | **Lack of Fit p-value** | **Adjusted R^2^** | **Predicted R^2^** |  |
| --- | --- | --- | --- | --- | --- |
| Linear | < 0.0001 | < 0.0001 | 0.7870 | 0.6438 |  |
| 2FI | 0.9618 | < 0.0001 | 0.7357 | 0.1650 |  |
| **Quadratic** | **< 0.0001** | **0.1472** | **0.9954** | **0.9771** | **Suggested** |
| Cubic | 0.1472 |  | 0.9973 |  | Aliased |

Table 2. Free amino acids – model fit summary

| **Source** | **Sequential p-value** | **Lack of Fit p-value** | **Adjusted R^2^** | **Predicted R^2^** |  |
| --- | --- | --- | --- | --- | --- |
| Linear | < 0.0001 | 0.0050 | 0.7957 | 0.7398 |  |
| 2FI | 0.9508 | 0.0024 | 0.7478 | 0.5406 |  |
| **Quadratic** | **0.0002** | **0.1791** | **0.9657** | **0.8371** | **Suggested** |
| Cubic | 0.1791 |  | 0.9778 |  | Aliased |

Table 3. TBARS – model fit summary

| **Source** | **Sequential p-value** | **Lack of Fit p-value** | **Adjusted R^2^** | **Predicted R^2^** |  |
| --- | --- | --- | --- | --- | --- |
| Linear | 0.2945 | 0.2387 | 0.0603 | -0.1149 |  |
| 2FI | 0.8782 | 0.1465 | -0.1274 | -0.6659 |  |
| **Quadratic** | **0.0121** | **0.8456** | **0.5770** | **0.3107** | **Suggested** |
| Cubic | 0.8456 |  | 0.4172 |  | Aliased |

Table 4. MDA – model fit summary

| **Source** | **Sequential p-value** | **Lack of Fit p-value** | **Adjusted R^2^** | **Predicted R^2^** |  |
| --- | --- | --- | --- | --- | --- |
| Linear | 0.1963 | 0.2432 | 0.1216 | -0.0670 |  |
| 2FI | 0.7927 | 0.1597 | -0.0215 | -0.5543 |  |
| **Quadratic** | **0.0190** | **0.7447** | **0.5687** | **0.1089** | **Suggested** |
| Cubic | 0.7447 |  | 0.4497 |  | Aliased |

Table 5. 2-Pentylfuran – model fit summary

| **Source** | **Sequential p-value** | **Lack of Fit p-value** | **Adjusted R^2^** | **Predicted R^2^** |  |
| --- | --- | --- | --- | --- | --- |
| Linear | < 0.0001 | 0.0002 | 0.9136 | 0.8532 |  |
| 2FI | 0.3107 | 0.0002 | 0.9195 | 0.7397 |  |
| **Quadratic** | **< 0.0001** | **0.1490** | **0.9959** | **0.9795** | **Suggested** |
| Cubic | 0.1490 |  | 0.9975 |  | Aliased |

Table 6. TVB-N – model fit summary

| **Source** | **Sequential p-value** | **Lack of Fit p-value** | **Adjusted R^2^** | **Predicted R^2^** |  |
| --- | --- | --- | --- | --- | --- |
| Linear | < 0.0001 | < 0.0001 | 0.7222 | 0.5343 |  |
| **2FI** | **0.0011** | **< 0.0001** | **0.9129** | **0.7506** | **Suggested** |
| Quadratic | 0.2702 | < 0.0001 | 0.9246 | 0.4330 |  |
| Cubic | < 0.0001 |  | 0.9999 |  | Aliased |

Table 7. DH - ANOVA for Quadratic model; BBD as mentioned in Table 1 in the manuscript

| **Source** | **Sum of Squares** | **df** | **Mean Square** | **F-value** | **p-value** | **Significance** |
| --- | --- | --- | --- | --- | --- | --- |
| **Model** | 1652.03 | 9 | 183.56 | 409.81 | < 0.0001 | significant |
| A-Temperature | 1176.12 | 1 | 1176.12 | 2625.77 | < 0.0001 | significant |
| B-Time | 171.12 | 1 | 171.12 | 382.05 | < 0.0001 | significant |
| C-Stirring | 18 | 1 | 18 | 40.19 | 0.0002 | significant |
| AB | 2.25 | 1 | 2.25 | 5.02 | 0.0553 | not significant |
| AC | 4 | 1 | 4 | 8.93 | 0.0174 | significant |
| BC | 1 | 1 | 1 | 2.23 | 0.1735 | not significant |
| A² | 242.73 | 1 | 242.73 | 541.92 | < 0.0001 | significant |
| B² | 54.73 | 1 | 54.73 | 122.2 | < 0.0001 | significant |
| C² | 2.73 | 1 | 2.73 | 6.11 | 0.0387 | significant |
| **Residual** | 3.58 | 8 | 0.4479 |  |  |  |
| Lack of Fit | 2.25 | 3 | 0.75 | 2.81 | 0.1472 | not significant |
| Pure Error | 1.33 | 5 | 0.2667 |  |  |  |
| **Cor Total** | 1655.61 | 17 |  |  |  |  |

The Model F-value of 409.81 implies the model is significant. There is only a 0.01% chance that an F-value this large could occur due to noise. P-values less than 0.0500 indicate model terms are significant. The Lack of Fit F-value of 2.81 implies the Lack of Fit is not significant relative to the pure error. There is a 14.72% chance that a "Lack of Fit F-value" this large could occur due to noise. Non-significant lack of fit is good - we want the model to fit.

Table 8. Free amino acids - ANOVA for Quadratic model; BBD as mentioned in Table 1 in the manuscript

| **Source** | **Sum of Squares** | **df** | **Mean Square** | **F-value** | **p-value** | **Significance** |
| --- | --- | --- | --- | --- | --- | --- |
| **Model** | 918.17 | 9 | 102.02 | 54.23 | < 0.0001 | significant |
| A-Temperature | 394.03 | 1 | 394.03 | 209.46 | < 0.0001 | significant |
| B-Time | 381.71 | 1 | 381.71 | 202.91 | < 0.0001 | significant |
| C-Stirring | 0.4797 | 1 | 0.4797 | 0.255 | 0.6272 | not significant |
| AB | 0.3209 | 1 | 0.3209 | 0.1706 | 0.6904 | not significant |
| AC | 1.41 | 1 | 1.41 | 0.7515 | 0.4112 | not significant |
| BC | 2.95 | 1 | 2.95 | 1.57 | 0.2459 | not significant |
| A² | 63.52 | 1 | 63.52 | 33.77 | 0.0004 | significant |
| B² | 47.52 | 1 | 47.52 | 25.26 | 0.001 | significant |
| C² | 5.67 | 1 | 5.67 | 3.01 | 0.1208 | not significant |
| **Residual** | 15.05 | 8 | 1.88 |  |  |  |
| Lack of Fit | 8.95 | 3 | 2.98 | 2.45 | 0.1791 | not significant |
| Pure Error | 6.1 | 5 | 1.22 |  |  |  |
| **Cor Total** | 933.22 | 17 |  |  |  |  |

The Model F-value of 54.23 implies the model is significant. There is only a 0.01% chance that an F-value this large could occur due to noise. P-values less than 0.0500 indicate model terms are significant. The Lack of Fit F-value of 2.45 implies the Lack of Fit is not significant relative to the pure error. There is a 17.91% chance that a "Lack of Fit F-value" this large could occur due to noise. Non-significant lack of fit is good - we want the model to fit.

Table 9. TBARS - ANOVA for Quadratic model; BBD as mentioned in Table 1 in the manuscript

| **Source** | **Sum of Squares** | **df** | **Mean Square** | **F-value** | **p-value** | **Significance** |
| --- | --- | --- | --- | --- | --- | --- |
| **Model** | 15489.76 | 9 | 1721.08 | 3.58 | 0.0433 | significant |
| A-Temperature | 101.18 | 1 | 101.18 | 0.2102 | 0.6588 | not significant |
| B-Time | 1030.81 | 1 | 1030.81 | 2.14 | 0.1815 | not significant |
| C-Stirring | 3240.93 | 1 | 3240.93 | 6.73 | 0.0319 | significant |
| AB | 547.09 | 1 | 547.09 | 1.14 | 0.3174 | not significant |
| AC | 59.99 | 1 | 59.99 | 0.1246 | 0.7332 | not significant |
| BC | 252.02 | 1 | 252.02 | 0.5237 | 0.4899 | not significant |
| A² | 3295.8 | 1 | 3295.8 | 6.85 | 0.0308 | significant |
| B² | 1386.85 | 1 | 1386.85 | 2.88 | 0.128 | not significant |
| C² | 3794.88 | 1 | 3794.88 | 7.89 | 0.0229 | significant |
| **Residual** | 3849.92 | 8 | 481.24 |  |  |  |
| Lack of Fit | 534.88 | 3 | 178.29 | 0.2689 | 0.8456 | not significant |
| Pure Error | 3315.04 | 5 | 663.01 |  |  |  |
| **Cor Total** | 19339.68 | 17 |  |  |  |  |

The Model F-value of 3.58 implies the model is significant. There is only a 4.33% chance that an F-value this large could occur due to noise. P-values less than 0.0500 indicate model terms are significant. The Lack of Fit F-value of 0.27 implies the Lack of Fit is not significant relative to the pure error. There is a 84.56% chance that a "Lack of Fit F-value" this large could occur due to noise. Non-significant lack of fit is good - we want the model to fit.

Table 10. MDA - ANOVA for Quadratic model; BBD as mentioned in Table 1 in the manuscript

| **Source** | **Sum of Squares** | **df** | **Mean Square** | **F-value** | **p-value** | **Significance** |
| --- | --- | --- | --- | --- | --- | --- |
| **Model** | 18.08 | 9 | 2.01 | 3.49 | 0.0462 | significant |
| A-Temperature | 2.75 | 1 | 2.75 | 4.78 | 0.0603 | not significant |
| B-Time | 0.0544 | 1 | 0.0544 | 0.0946 | 0.7663 | not significant |
| C-Stirring | 3.47 | 1 | 3.47 | 6.03 | 0.0396 | significant |
| AB | 1.22 | 1 | 1.22 | 2.12 | 0.1834 | not significant |
| AC | 0.0225 | 1 | 0.0225 | 0.0391 | 0.8482 | not significant |
| BC | 0.1722 | 1 | 0.1722 | 0.2992 | 0.5993 | not significant |
| A² | 3.69 | 1 | 3.69 | 6.42 | 0.0351 | significant |
| B² | 1.19 | 1 | 1.19 | 2.07 | 0.1882 | not significant |
| C² | 3.73 | 1 | 3.73 | 6.49 | 0.0344 | significant |
| **Residual** | 4.61 | 8 | 0.5757 |  |  |  |
| Lack of Fit | 0.933 | 3 | 0.311 | 0.4234 | 0.7447 | not significant |
| Pure Error | 3.67 | 5 | 0.7345 |  |  |  |
| **Cor Total** | 22.69 | 17 |  |  |  |  |

The Model F-value of 3.49 implies the model is significant. There is only a 4.62% chance that an F-value this large could occur due to noise. P-values less than 0.0500 indicate model terms are significant. The Lack of Fit F-value of 0.42 implies the Lack of Fit is not significant relative to the pure error. There is a 74.47% chance that a "Lack of Fit F-value" this large could occur due to noise. Non-significant lack of fit is good - we want the model to fit.

Table 11. 2-Pentylfuran - ANOVA for Quadratic model; BBD as mentioned in Table 1 in the manuscript

| **Source** | **Sum of Squares** | **df** | **Mean Square** | **F-value** | **p-value** | **Significance** |
| --- | --- | --- | --- | --- | --- | --- |
| **Model** | 1691.07 | 9 | 187.9 | 456.36 | < 0.0001 | significant |
| A-Temperature | 1198.05 | 1 | 1198.05 | 2909.8 | < 0.0001 | significant |
| B-Time | 349.8 | 1 | 349.8 | 849.59 | < 0.0001 | significant |
| C-Stirring | 25.92 | 1 | 25.92 | 62.95 | < 0.0001 | significant |
| AB | 32.26 | 1 | 32.26 | 78.36 | < 0.0001 | significant |
| AC | 0.0484 | 1 | 0.0484 | 0.1176 | 0.7405 | not significant |
| BC | 0.01 | 1 | 0.01 | 0.0243 | 0.88 | not significant |
| A² | 54.48 | 1 | 54.48 | 132.31 | < 0.0001 | significant |
| B² | 30.45 | 1 | 30.45 | 73.96 | < 0.0001 | significant |
| C² | 7.68 | 1 | 7.68 | 18.65 | 0.0025 | significant |
| **Residual** | 3.29 | 8 | 0.4117 |  |  |  |
| Lack of Fit | 2.06 | 3 | 0.6872 | 2.79 | 0.149 | not significant |
| Pure Error | 1.23 | 5 | 0.2464 |  |  |  |
| **Cor Total** | 1694.36 | 17 |  |  |  |  |

The Model F-value of 456.36 implies the model is significant. There is only a 0.01% chance that an F-value this large could occur due to noise. P-values less than 0.0500 indicate model terms are significant. The Lack of Fit F-value of 2.79 implies the Lack of Fit is not significant relative to the pure error. There is a 14.90% chance that a "Lack of Fit F-value" this large could occur due to noise. Non-significant lack of fit is good - we want the model to fit.

Table 12. TVB-N - ANOVA for Quadratic model; BBD as mentioned in Table 1 in the manuscript

| **Source** | **Sum of Squares** | **df** | **Mean Square** | **F-value** | **p-value** | **Significance** |
| --- | --- | --- | --- | --- | --- | --- |
| **Model** | 470.55 | 9 | 52.28 | 24.18 | < 0.0001 | significant |
| A-Temperature | 275.42 | 1 | 275.42 | 127.35 | < 0.0001 | significant |
| B-Time | 83.79 | 1 | 83.79 | 38.74 | 0.0003 | significant |
| C-Stirring | 17.02 | 1 | 17.02 | 7.87 | 0.023 | significant |
| AB | 64.16 | 1 | 64.16 | 29.67 | 0.0006 | significant |
| AC | 18.06 | 1 | 18.06 | 8.35 | 0.0202 | significant |
| BC | 1.89 | 1 | 1.89 | 0.8742 | 0.3771 | not significant |
| A² | 8.04 | 1 | 8.04 | 3.72 | 0.09 | significant |
| B² | 1.27 | 1 | 1.27 | 0.5875 | 0.4654 | not significant |
| C² | 0.0068 | 1 | 0.0068 | 0.0032 | 0.9565 | not significant |
| **Residual** | 17.3 | 8 | 2.16 |  |  |  |
| Lack of Fit | 17.29 | 3 | 5.76 | 2136.92 | < 0.0001 | significant |
| Pure Error | 0.0135 | 5 | 0.0027 |  |  |  |
| **Cor Total** | 487.85 | 17 |  |  |  |  |

The Model F-value of 24.18 implies the model is significant. There is only a 0.01% chance that an F-value this large could occur due to noise. P-values less than 0.0500 indicate model terms are significant. The Lack of Fit F-value of 2136.92 implies the Lack of Fit is significant. There is only a 0.01% chance that a Lack of Fit F-value this large could occur due to noise. Significant lack of fit is bad - we want the model to fit.


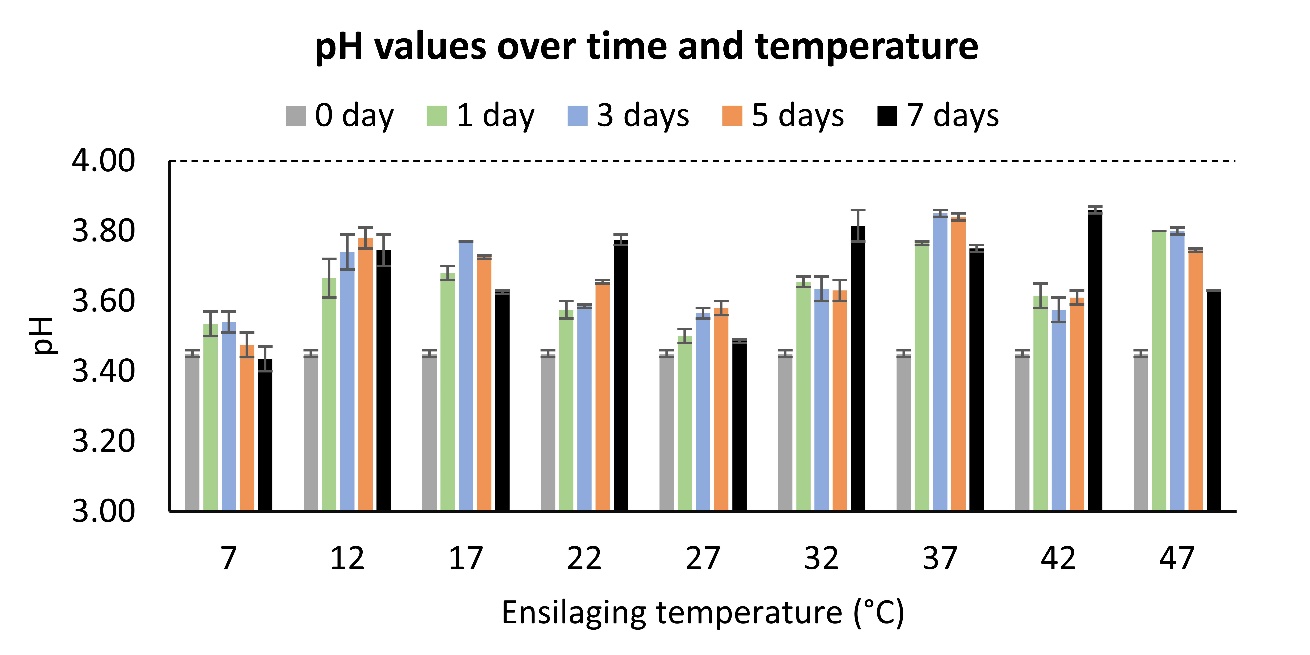


Figure 1. pH values in herring by-product silages as a function of ensilaging time and temperature. Day-0 pH refers to pH after addition of acid.

Figure 2. Effect of temperature and time on DH in herring by-product silage.


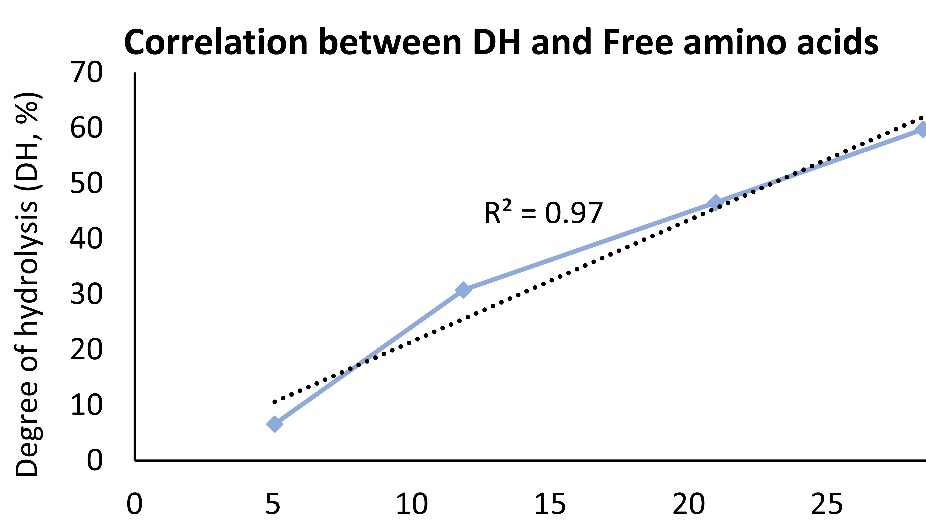


Figure 3. Correlation between DH and free amino acids formation when ensilaging was performed at 22°C using herring by-products from Autumn 2017.


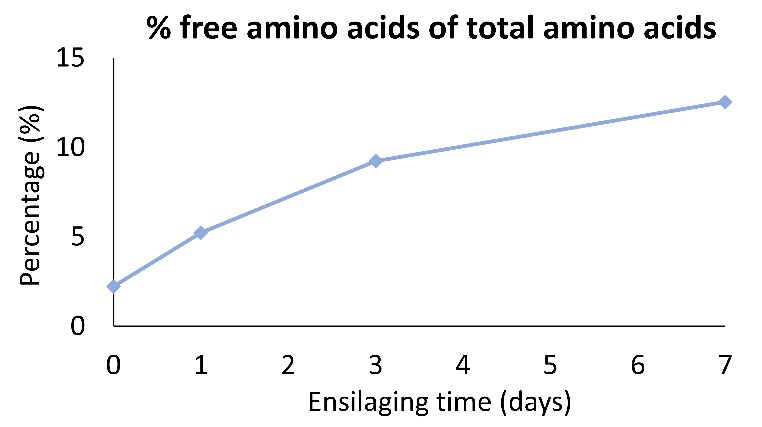


Figure 4. Percentage of free amino acids compared to total amino acids when ensilaging was performed at 22°C using herring by-products from Autumn 2017.


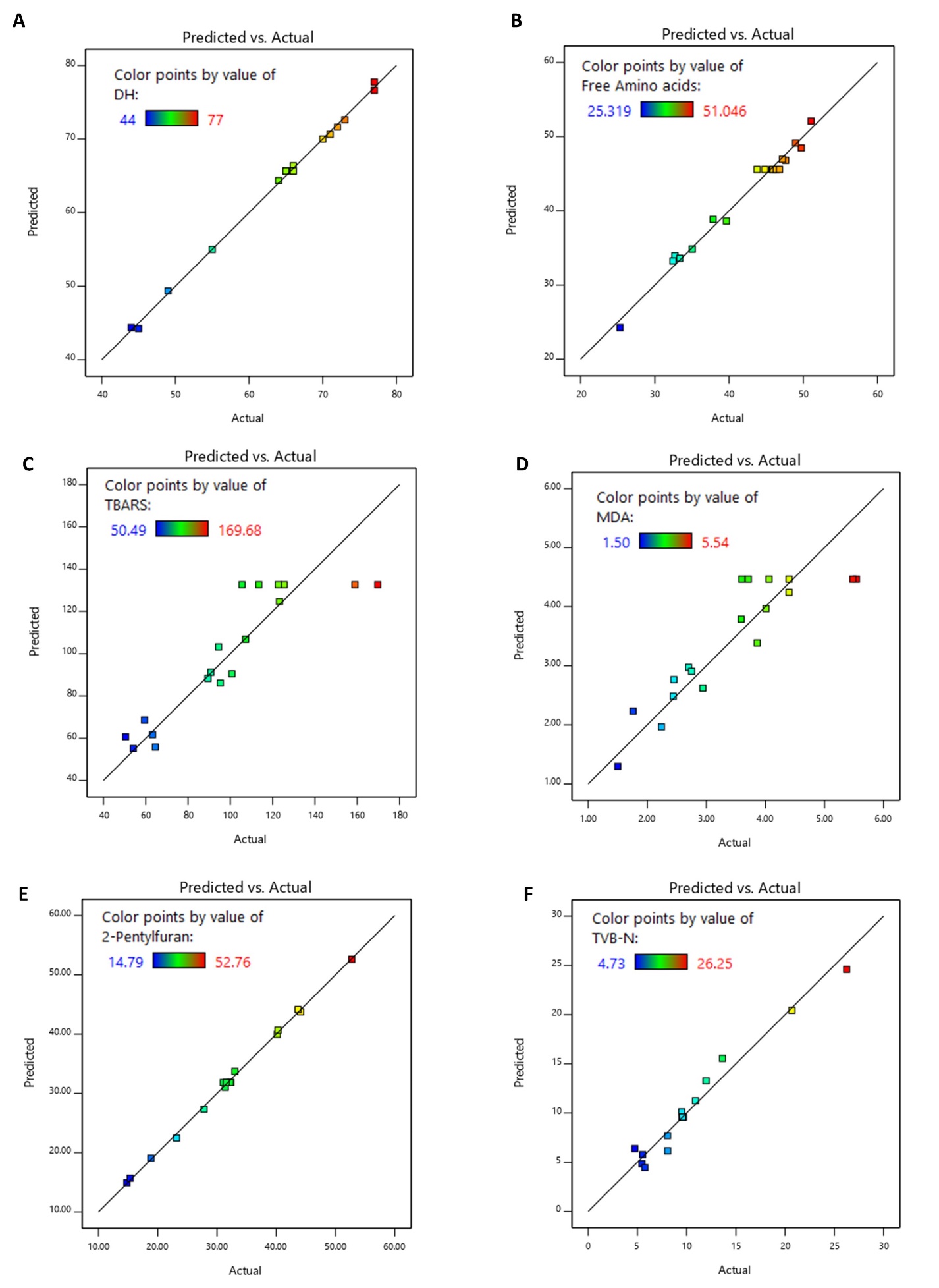


Figure 5. Relationship between predicted vs actual responses linked to the experimental design (BBD) as mentioned in Table 1 in the manuscript; (A) DH, (B) Free amino acids, (C) TBARS, (D) MDA, (E) 2-Pentylfuran, and (F) TVB-N.


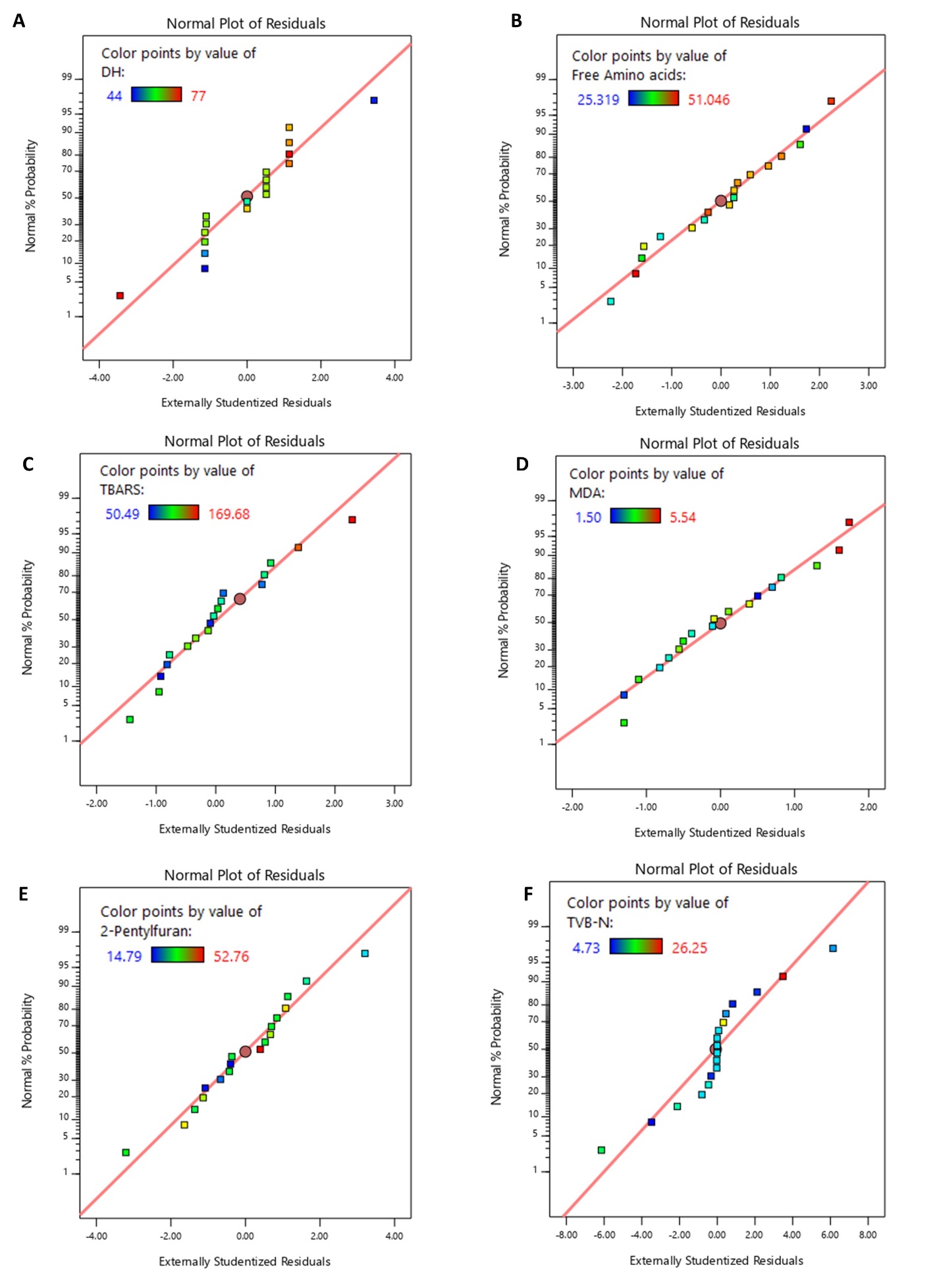


Figure 6. Normal plot of residuals for studied responses linked to the experimental design (BBD) as mentioned in Table 1 in the manuscript; (A) DH, (B) Free amino acids, (C) TBARS, (D) MDA, (E) 2-Pentylfuran, and (F) TVB-N.


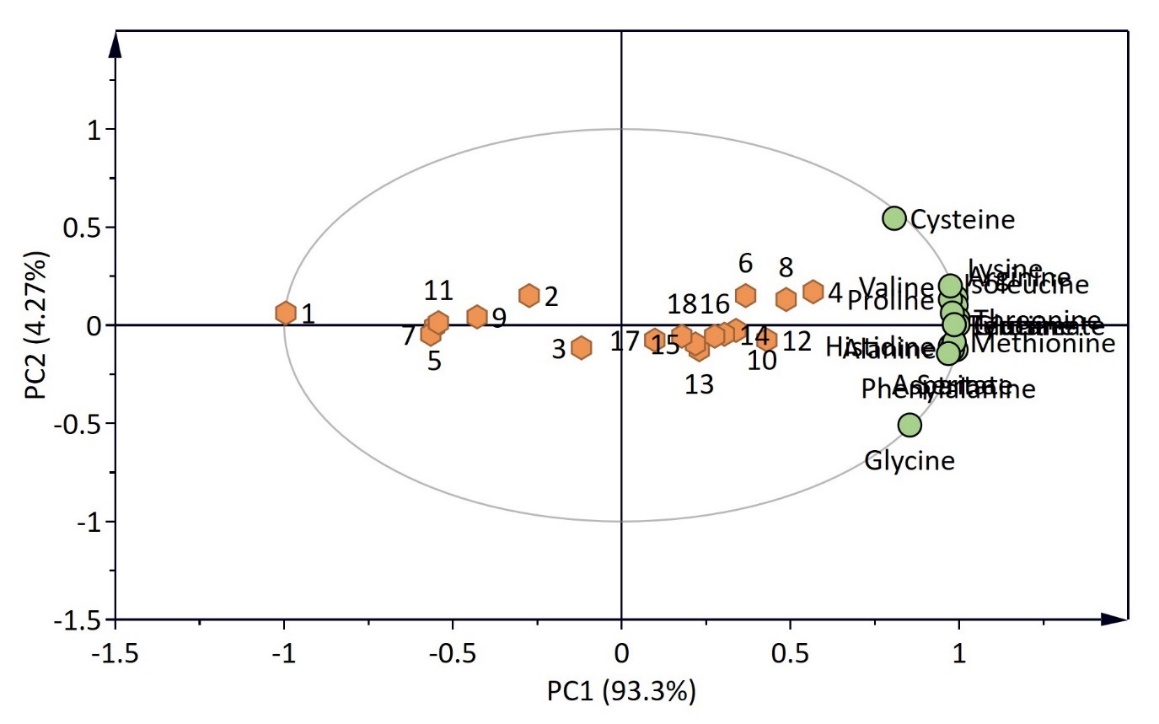


Figure 7. PCA bi-plot showing an overview of specific free amino acids formed during ensilaging trials linked to the experimental design (BBD) as mentioned in Table 1 in the manuscript. Most of the free amino acids were highly correlated (see correlation matrix in the next figure) except cysteine and glycine.


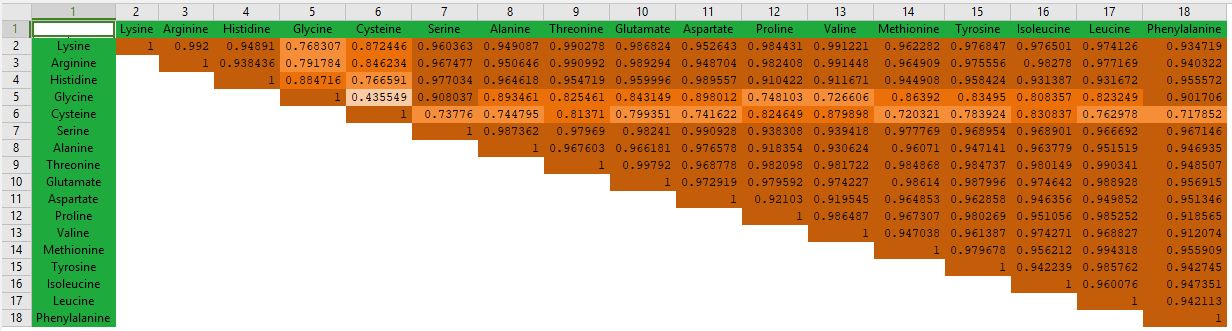


Figure 8. Correlation matrix of free amino acids formed during trials linked to the experimental design (BBD) as mentioned in Table 1 in the manuscript. A value close to 1 represents high correlation among free amino acids.
